# Porcine β-Defensin 114: Creating a Dichotomous Response to Inflammation

**DOI:** 10.3390/ijms25021016

**Published:** 2024-01-13

**Authors:** Guoqi Su, Sheng Huang, Shan Jiang, Li Chen, Feiyun Yang, Zuohua Liu, Guixue Wang, Jinxiu Huang

**Affiliations:** 1 Chongqing Academy of Animal Sciences, Chongqing 402460, China; master2015@163.com (G.S.); chenliyouxiang8@163.com (L.C.);; 2 National Pig Technology Innovation Center, Chongqing 402460, China; 3 Key Laboratory of Biorheological Science & Technology, Ministry of Education, State & Local Joint Engineering Laboratory for Vascular Implants, College of Bioengineering, Chongqing University, Chongqing 402460, China

**Keywords:** RNA-seq, RAW264.7, mice, lipopolysaccharide, inflammatory response

## Abstract

The immunity-related functions of defensins seem to be dependent on environmental stimuli, the cell type, and the concentration of peptides. However, the function and mechanism of porcine β-defensin 114 (pBD114) in regulating the inflammatory response to macrophages are unclear. Therefore, the modulatory effects of porcine pBD114 on the inflammatory response were investigated by treating the mouse monocyte macrophage cell line RAW264.7 with different concentrations of pBD114 with or without lipopolysaccharide (LPS). RNA-seq analysis was performed to investigate the mechanisms underlying pBD114’s regulation of inflammatory responses in macrophages. In addition, the inflammatory response-modulating effects of pBD114 were also further verified with a mouse assay. The results showed that 100 μg/mL of pBD114 significantly promoted the secretion of TNF-α and IL-10 in RAW264.7. However, the LPS-induced increase in TNFα in the RAW264.7 cell cultures was significantly decreased with 10 μg/mL of pBD114. These results suggest that pBD114 can exhibit pro-inflammatory activities under normal physiological conditions with 100 μg/mL of pBD114, and anti-inflammatory activities during an excessive inflammatory response with 10 μg/mL of pBD114. RNA-seq analysis was performed to gain further insights into the effects of pBD114 on the inflammatory response. Among the pBD114-promoting RAW264.7 pro-inflammatory responses, pBD114 significantly up-regulated 1170 genes and down-regulated 724 genes. KEGG enrichment showed that the differentially expressed genes (DEGs) were significantly enriched in the immune- and signal-transduction-related signaling pathways. Protein-Protein Interaction (PPI) and key driver analysis (KDA) analyses revealed that *Bcl10* and *Bcl3* were the key genes. In addition, pBD114 significantly up-regulated 12 genes and down-regulated 38 genes in the anti-inflammatory response. KEGG enrichment analysis revealed that the DEGs were mainly enriched in the “Cytokine–cytokine receptor interaction” signaling pathway, and PPI and KDA analyses showed that *Stat1* and *Csf2* were the key genes. The results of qRT-PCR verified those of RNA-seq. In vivo mouse tests also confirmed the pro- or anti-inflammatory activities of pBD114. Although the inflammatory response is a rapid and complex physiological reaction to noxious stimuli, this study found that pBD114 plays an essential role mainly by acting on the genes related to immunity, signal transduction, signaling molecules, and interactions. In conclusion, this study provides a certain theoretical basis for the research and application of defensins.

## 1. Introduction

Inflammation is a response triggered by damage to living tissues. The inflammatory response is part of the innate defense mechanism of the body against infectious or non-infectious etiologies [1]. A normal inflammatory response is characterized by the temporally restricted up-regulation of inflammatory activity that occurs when a threat is present and that resolves once the threat has passed. However, systemic chronic inflammation (SCI) can cause the breakdown of immune tolerance and lead to significant alterations in all the tissues and organs, as well as normal cellular physiology, which can increase the risk of young and older individuals contracting various non-communicable diseases [2]. In humans, SCI has been linked to diseases such as cancer, heart disease, diabetes, arthritis, depression, and Alzheimer’s [3]. Similarly, many pro-inflammatory mediators have been associated with an enhanced resistance to a range of important poultry and pig pathogens in livestock animals. However, inflammation may have undesirable consequences, including potentially exacerbating tissue damage and diverting the nutrients from productive purposes [4,5,6]. These detrimental effects underscore the necessity for a delicate balance in the inflammatory response; when this balance is disrupted, it can have severe implications for human and animal health [7,8].

Defensins are a family of cationic antimicrobial peptides active against various infectious microbes, including bacteria, viruses, and fungi, that play important roles as innate effectors and immune modulators in the control of microbial infection [9,10,11]. They function primarily as antimicrobial peptides, eliminating potential pathogens by disrupting their cellular membranes [12]. However, their role extends beyond direct antimicrobial activity [13,14]. Defensins display substantial immunomodulatory properties in vitro and in vivo, and these features are becoming increasingly appreciated in the literature [10,15,16,17]. Our previous studies showed that pBD114 suppresses endotoxin-induced inflammation and apoptosis in IECs through the down-regulation of two critical inflammation-associated signaling proteins, NF-kappa-B inhibitor alpha (IkB-α) and extracellular signal-regulated kinase1/2 (ERK1/2); it also suppresses inflammation and IEC apoptosis in mice exposed to bacterial endotoxins [18]. Whether in the assays of IPEC-J2 cell [19] or mice [20], porcine β defensin 129 (pBD129) could alleviate LPS-induced inflammatory responses by reducing the expression of inflammatory factors, and additionally, pBD129 could enhance the expression of tight junction proteins and reduced the rate of apoptosis in intestinal epithelial cells. Similarly, pBD2 also could decrease the rates of cell permeability and apoptosis, reduce the inflammatory infiltration of Caco-2 cells, and reduce the expression of DSS-induced inflammatory factors in mouse colons via AKT and NF-κB [21]. Synthetic porcine β defensin 2 (pBD2) decreased the expression of *TNFα*, *IL-1β*, and *IL-8* in piglet intestinal epithelial cells [22]. Hence, porcine defensins are important in maintaining intestinal health and reducing the inflammation of intestinal epithelial cells.

**Figure 1:**
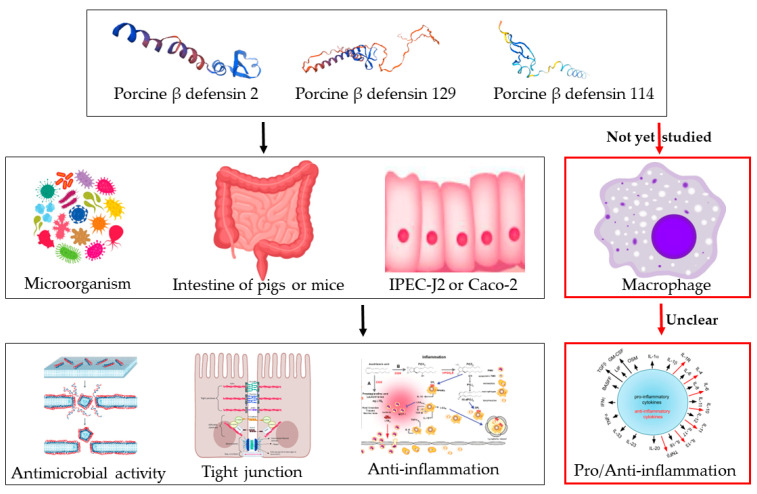
Biological functions of porcine defensins. It has been shown that porcine β-defensins have some antimicrobial activity, and porcine β-defensins 2/129 can enhance the intestinal barrier and inhibit inflammation in the mouse intestine, IPEC-J2, or Caco-2 cells. However, the inflammatory response of porcine defensins to macrophages has not yet been studied, and the mechanism is still unclear.

Porcine defensins have been shown to have antibacterial [23], antiviral [24], antioxidant [25,26], intestinal barrier maintenance [25], and anti-inflammatory effects [27] (Figure 1), but it is not clear whether porcine defensins possess the ability to activate inflammatory responses. Our previous studies have shown that pBD114 modulates inflammation, barrier processes, and apoptosis in porcine intestinal epithelial cells [18]. Studies have shown that defensins’ functions seem to depend on the environmental stimuli, cell and tissue types, interactions with different cellular receptors, and the concentration of the peptides [28]. For instance, the pro- and anti-inflammatory effects of cathelicidin LL-37 are concentration-dependent, i.e., the former are visible at >20 µg/mL, whereas the latter are visible at 1–5 µg/mL [29].We supposed that pBD114 plays different inflammatory regulatory roles under different environmental stimuli. Therefore, in this study, we investigated the effects of pBD114 on the inflammatory responses by using in vivo and in vitro assays. Our study contributes to understand the function and mechanism of porcine defensins in balancing the inflammation of host cells.

## 2. Results

### 2.1. Preparation and Basic Biological Characteristics of pBD114

**Figure 2:**
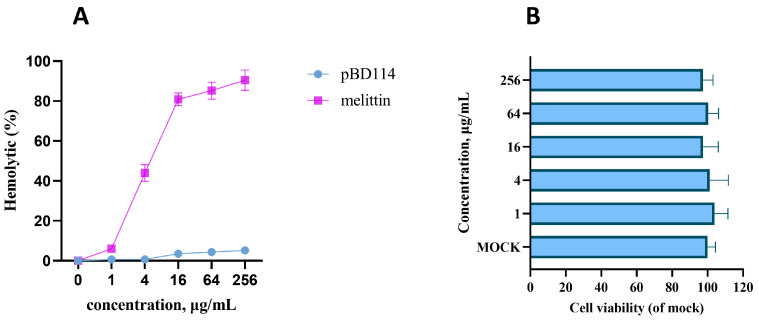
The hemolytic value (**A**) of pBD114 was measured using 4% porcine erythrocytes, which were incubated with 1, 4, 16, 64, and 256 μg/mL pBD114 and melittin for 1 h at 37 °C. RAW264.7 was treated with pBD114 at final concentrations of 0 (MOCK), 1, 4, 16, 64, and 256 μg/mL for 24 h, and then the cytotoxicity (**B**) of pBD114 was determined using CCK-8 according to the user’s guide.

The SignalP-5.0 online analysis results in Figure S1A show that there is a significant signal peptide cutting site between the 22nd and 23rd amino acids. Hence, the mature peptide of pBD114 is 47-amino-acid-long. The mature peptide of pBD114 was synthesized with artificial chemical solid-phase synthesis technology. The purity was >95% with RP-HPLC (Figure S1B,C), and the molecular mass was confirmed to be 5552.47 by electrospray ionization-mass spectrometry (ESI-MS) (Figure S1D). The bioactivity of pBD114 was predicted online with AMPfun; the results indicated that targeting Gram-positive and -negative bacteria yielded a negative prediction (Figure S2). The antimicrobial activity of pBD114 against *Staphylococcus aureus*, *Enterococcus faecalis*, *Escherichia coli*, *Pseudomonas aeruginosa*, *Salmonella typhimurium*, and *Klebsiella pneumoniae* was explored using the minimum inhibitory concentration (MIC). The MIC values of them all were more than 128 μg/mL (Table S1), indicating that the antibacterial activity level of pBD114 was low. The hemolysis of pBD114 predicted online with HemoPI first, and the result indicated that pBD114 is not hemolytic (Figure S3). The hemolytic test conducted with a 4% rabbit red blood cell suspension showed that when the concentration of pBD114 was increased by 256 μg/mL, the hemolytic rate was still no more than 5% (Figure 2A), indicating that pBD114 is less hemolytic and safe. RAW264.7 was also used to examine the cytotoxicity of pBD114, and cell viability was measured using CCK-8 after a treatment with different concentrations of pBD114. The cell viability was not affected by pBD114, indicating that pBD114 has no cytotoxicity (Figure 2B). These results suggested that pBD114 is safe at a concentration of no more than 256 μg/mL.

### 2.2. Pro-Inflammatory and Anti-Inflammatory Activity of pBD114 on the Mouse Mononuclear Macrophage RAW264.7 Cells

**Figure 3:**
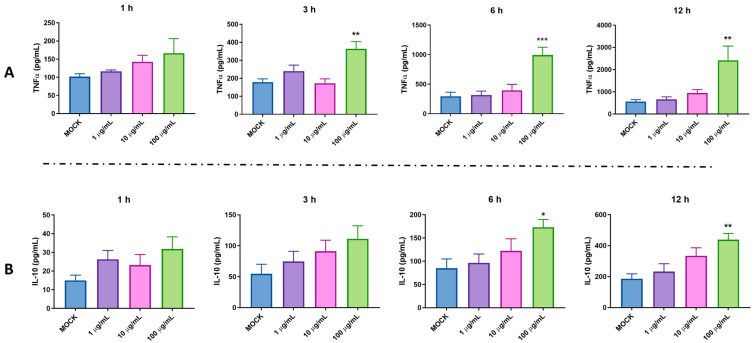
The pro-inflammatory activity of pBD114 on the RAW264.7 cells. The RAW264.7 cells were treated with 0 (MOCK), 1, 10, and 100 μg/mL pBD114 for 1, 3, 6, and 12 h, respectively, and the concentrations of TNFα (**A**) and IL-10 (**B**) in the supernatant of cell culture were detected using ELISA. The significance levels are (relative to MOCK) * *p* < 0.05, ** *p* < 0.01, and *** *p* < 0.001.

**Figure 4:**
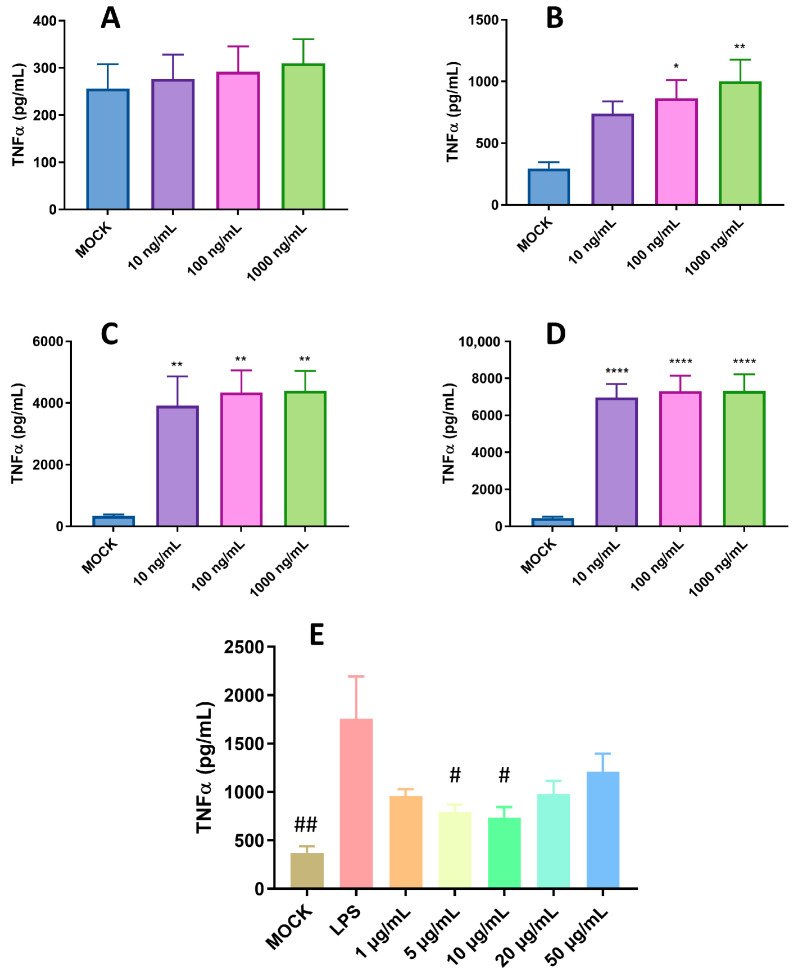
Anti-inflammatory activity of pBD114 on the RAW264.7 cells. The RAW264.7 cells were treated with 0 (MOCK), 10, 100, and 1000 ng/mL LPS for 0.5, 1, 2, and 4 h; the concentration of TNFα ((**A**) 0.5 h; (**B**) 1 h; (**C**) 2 h; and (**D**) 4 h) in the supernatant of cell culture was detected using ELISA. RAW264.7 cells were co-cultured with 1, 5, 10, 20, and 50 μg/mL pBD114 and 10 ng/mL LPS for 2 h, and the concentration of TNFα (**E**) in the supernatant of cell culture was detected using ELISA. The significance levels are (relative to MOCK) * *p* < 0.05, ** *p* < 0.01, and **** *p* < 0.0001. The significance levels are (relative to LPS) # *p* < 0.05, and ## *p* < 0.01.

Our previous study indicated that pBD114 is essential in maintaining intestinal epithelium homeostasis in response to various infections or diseases [18]. However, the effects of pBD114 on the inflammatory response to macrophages remain unclear. As shown in Figure 3, 100 μg/mL pBD114 co-cultured with RAW264.7 cells for 3, 6, and 12 h (Figure 3A) significantly increased the concentration of TNFα in the culture medium (*p* < 0.05). The concentration of IL-10 in the culture medium increased considerably after the co-culturing of 100 μg/mL pBD114 with RAW264.7 cells for 6 and 12 h (Figure 3B) (*p* < 0.05). As shown in Figure 4, 100 and 1000 ng/mL LPS treated for 1 h (Figure 4B) could induce the inflammation in the RAW264.7 cells, and significantly increase the protein concentration of the inflammatory factor TNF-α in the cell culture medium (*p* < 0.05). The treatment with 10, 100, and 1000 ng/mL of LPS for 2 (Figure 4C) and 4 h (Figure 4C) could significantly induce the protein concentration of the inflammatory factor TNF-α in the RAW264.7 cell culture medium of mouse mononuclear macrophages (*p* < 0.05). Therefore, in the follow-up experiment, the immune activation of RAW264.7 cells was performed with 10 ng/mL LPS for 2 h. To study the inhibitory effect of pBD114 on the immune activation of RAW264.7 cells, in this study, the RAW264.7 cells were co-cultured with 1, 5, 10, 20, and 50 μg/mL pBD114 and 10 ng/mL LPS for 2 h. The results indicated that 5 and 10 μg/mL pBD114 (Figure 4E) significantly inhibited the production of the inflammatory cytokine TNF-α (*p* < 0.05). The results showed that pBD114 (5 and 10 μg/mL) possessed immunosuppressive activity in the RAW264.7 cells activated by LPS.

### 2.3. RNA-Seq Analyzed the Mechanism of pBD114 on the Inflammation of the Mouse Mononuclear Macrophage RAW264.7 Cells

The effects of pBD114 on the inflammatory response to the RAW246.7 cells and the underlying mechanism were analyzed in depth by using RNA-seq. Sixteen samples were measured using the DNBSEQ platform, with an average output of 1.17G data per sample. The average matching rate of the samples against the genome was 90.06%, and the average matching rate against the gene set was 84.86%; a total of 18,126 genes were detected. After quality filtering and trimming, 21.20 to 23.86 M clean reads were obtained, and the percentage of the Q20 and Q30 bases was over 97.40% and 92.22%, respectively (Table S2). The obtained clear reads were mapped with the reference genome (genome ref. number: GCF_000001635.26_GRCm38.p6) using HISAT2 (v2.1.0) software, and the mapping rate was higher than 88.51% (Table S3).

#### 2.3.1. Differentially Expressed Genes

**Figure 5:**
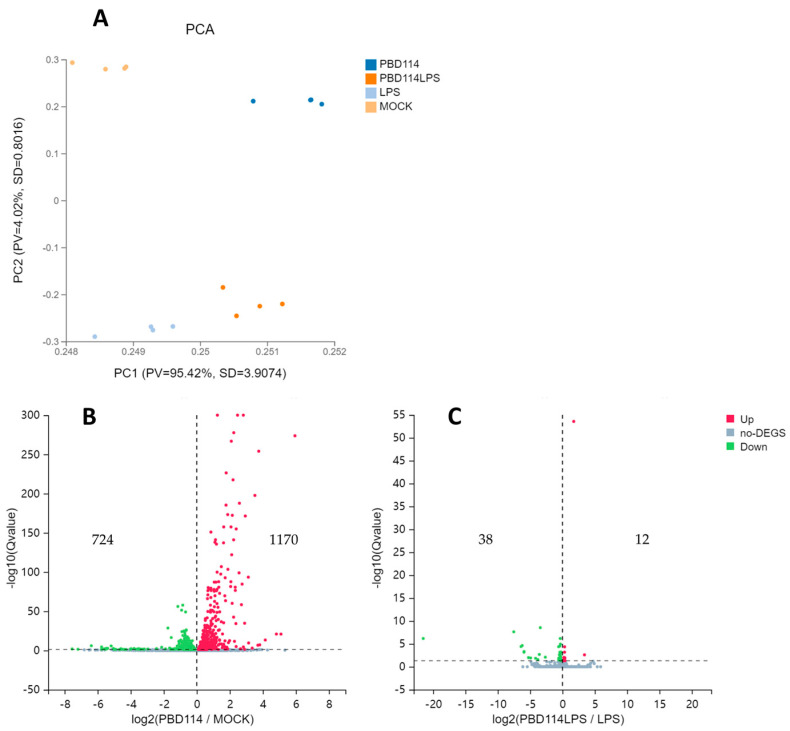
Analysis of differential expression genes (DEGs). (**A**) Principal component analysis (PCA). The X and Y axes represent the new dataset corresponding to the principal components obtained after dimensionality reduction in sample expressions, which were used to represent the disparity between the samples; values in parentheses in the axes’ labels represent the percentage of the overall variance explained by the corresponding principal components. Dots represent each sample, and the same color represents the same sample group. pv denotes proportion of variance, and SD denotes standard deviation. Volcano plots of PBD114/MOCK (**B**) and PBD114LPS/LPS (**C**). The X axis represents log2-transformed difference multiplicity values, and the Y axis represents −log10-transformed significance values.

Prior to the analysis of DEGs, principal component analysis (PCA) was performed. The PCA plots show significant differences between the four groups (Figure 5A). From the volcano plots of PBD114/MOCK (Figure 5B), there are 1894 DEGs, including 1170 up-regulated genes and 724 down-regulated genes. From the volcano plots of PBD114LPS/LPS (Figure 5C), there are 50 DEGs, including 12 up-regulated genes and 38 down-regulated genes. Detailed DEGs data are shown in Table S4.

#### 2.3.2. KEGG Enrichment Analysis

**Figure 6:**
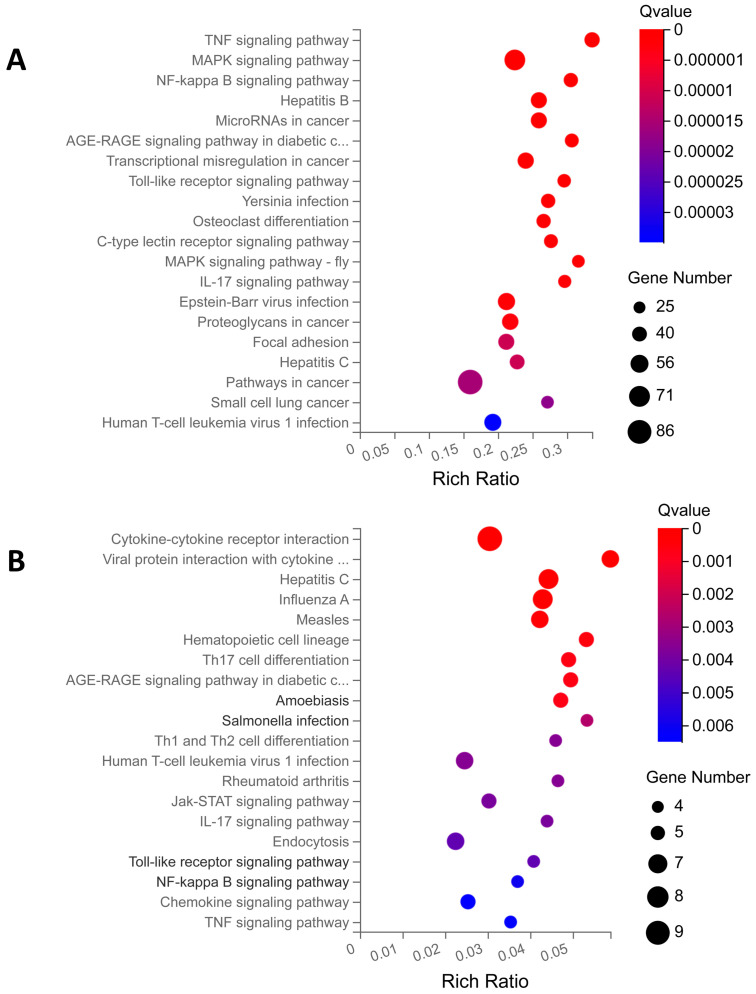
KEGG enrichment analysis of differential expression genes (DEGs) for PBD114/MOCK (**A**) and PBD114LPS/LPS (**B**). The X axis is the enrichment ratio (the ratio of the number of genes annotating an entry in a selected gene set to the total number of genes annotating that entry in the species, which is calculated as Rich Ratio = Term Candidate Gene Num/Term Gene Num). The Y axis is the KEGG Pathway, and the size of the bubbles indicates the number of genes annotated to a certain KEGG. The color represents the enrichment significance value (*q*-value or *p*-value; see the legend for details). The redder the color is, the smaller the significance value is.

KEGG enrichment is based on KEGG pathway annotation classification, the functional classification of differential genes, and enrichment analysis using the phyper function in R v3.4.4 software. By analyzing the KEGG pathway enrichment of DEGs of PBD114/MOCK, a total of 329 signaling pathways were enriched using KEGG pathway enrichment analysis, of which 95 KEGG pathways were significantly enriched (Q ≤ 0.05); detailed data are shown in Table S5. The analysis of the first 20 enriched pathways showed the presence of three immune-related pathways, the Toll-like receptor, C-type lectin receptor, and IL-17 signaling pathways. It was noted that the C-type lectin receptor signaling pathway with the most significant number of DEGs was enriched (Figure 6A). At the same time, the analysis of the first 20 enriched pathways showed the presence of three signal transduction-related pathways, the TNF, MAPK, and NF-kappa B signaling pathways (-fly); it was noted that the MAPK signaling pathway with the most significant number of DEGs was enriched (Figure 5A). The DEGs of PBD114LPS/LPS were analyzed using KEGG pathway enrichment, and a total of 117 signaling pathways were enriched, of which 30 KEGG pathways were significantly enriched (Q ≤ 0.05); detailed data are shown in Table S5. The top 20 pathways with the most significant enrichments related to immunomodulation were selected and are presented in the form of bubble plots (Figure 6B). The Hematopoietic cell lineage, Th17 cell differentiation, Th1 and Th2 cell differentiation, IL-17 signaling pathway, Toll-like receptor signaling pathway, and Chemokine signaling pathway related immune-system are among them; it was noted that the Hematopoietic cell lineage with the largest number of DEGs was enriched. Among cytokine–cytokine receptor interaction, viral protein interaction with the cytokine and cytokine receptor, the Jak-STAT, NF-kappa B, Chemokine signaling pathways, and TNF signaling pathway related signal transduction, it was noted that the cytokine–cytokine receptor interaction with the largest number of DEGs was enriched.

#### 2.3.3. PPI and KDA Analyses

**Figure 7:**
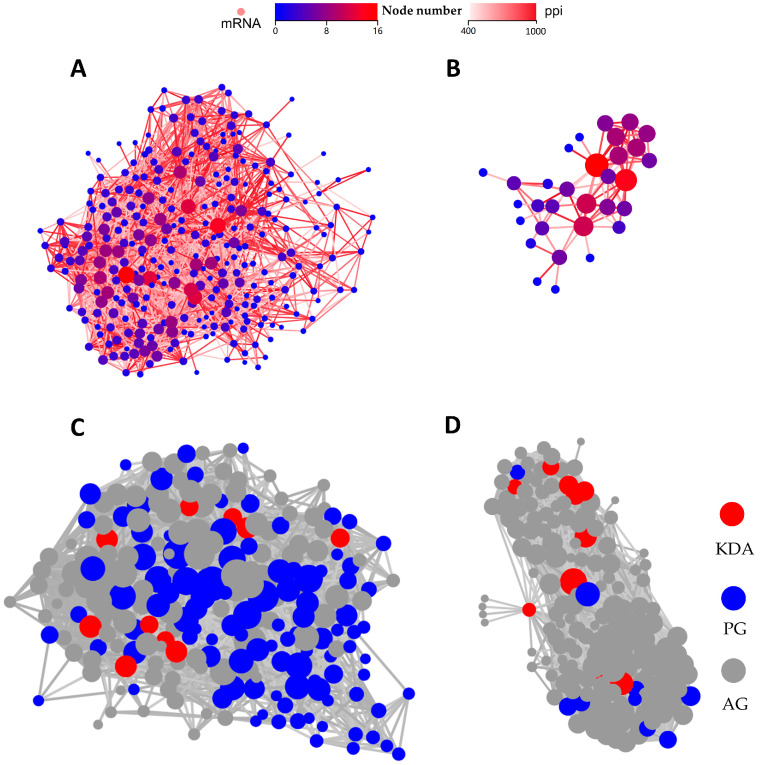
PPI and KDA analyses of differential expression genes (DEGs) for PBD114/MOCK (**A**,**C**) and PBD114LPS/LPS (**B**,**D**).

PPI and KDA analyses were performed to further explore the key genes of pBD114 regulating the RAW264.7 cells inflammatory response. Thress hundred and three DEGs significantly enriched by PBD114/MOCK in 28 signaling pathways related to immunity and signaling were selected for analysis, and the PPI results showed that *Tnf*, *Kras*, *Jun*, *Src*, and *Mapk14* had the most interactions with the other proteins (Figure 7A). Further KDA analysis identified *Bcl10*, *Bcl3*, *Cflar*, *Il1a*, *Dusp1*, *Traf1*, *Map2k7*, *Map3k8*, *Irf5*, and *Ikbke* as the key driver genes (Figure 7C). These DEGs were predominantly enriched in the NF-kappa B, C-type lectin receptor, MAPK, and Toll-like receptor signaling pathways (Table S6). Since there were only 50 DEGs in PBD114LPS/LPS, we subjected all DEGs to PPI and DEGs analyses. The results showed that *Stat1* and *Cxcl10* had the most interactions with the other proteins (Figure 7B). Further KDA analyses showed that *Stat1*, *Csf2*, and *Il1b* are the key driver genes (Figure 7D). These DEGs were mainly enriched in the C-type lectin receptor, MAPK, and Chemokine signaling pathways (Table S6).

#### 2.3.4. Validation of RNA-Seq Results Via Quantitative PCR Analysis

**Figure 8:**
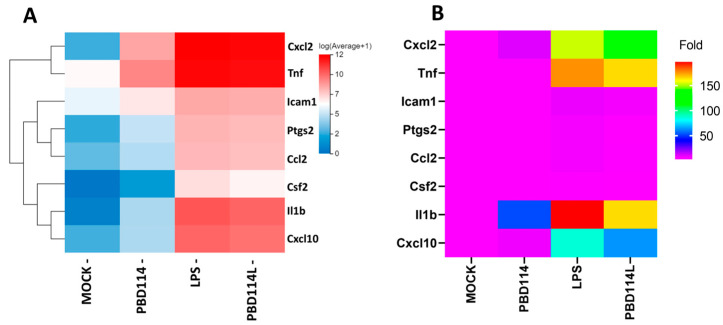
Validation of RNA-seq (**A**) data using real-time qRT-PCR (**B**).

Eight genes related to the inflammatory response were selected upon performing qRT-PCR to further validate the RNA sequencing data (Figure 8B). The results showed that the expression trend of each gene was consistent with the high-throughput sequencing data (Figure 8A).

### 2.4. Modulation of Inflammatory Responses in Mice by pBD114

**Figure 9:**
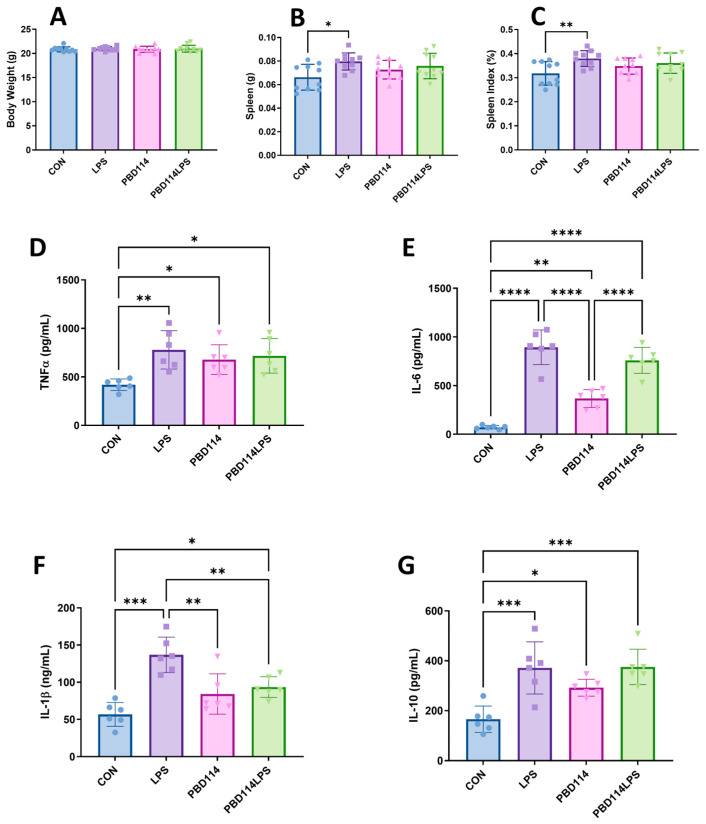
Effects of pBD114 on spleen and serum inflammatory factors in mice. The intraperitoneal injections of LPS (5.0 mg/kg) and pBD114 (5.0 mg/kg) were administered in mice for 12 h. Their body weight (**A**), spleen weight (**B**), and spleen index (**C**) were calculated, and the concentrations of the TNFα (**D**), IL-6 (**E**), IL-1β (**F**), and IL-10 (**G**) proteins in the mice serum were measured by ELISA. The significance levels are * *p* < 0.05, ** *p* < 0.01, *** *p* < 0.001, and **** *p* < 0.0001.

The in vitro results of this study showed that pBD114 is able to modulate the inflammatory response of the RAW264.7 cells. In this study, we investigated the effects of pBD114 on the inflammatory response in mice via the intraperitoneal injection of pBD114 and LPS administered for 12 h, to verify whether pBD114 could perform the same biological function in vivo. As shown in Figure 9, the weight of the mice was not affected by pBD114 and LPS (Figure 9A), but LPS significantly increased the weight of the spleen (Figure 9B) and the spleen index (Figure 9C) (*p* < 0.05). LPS significantly increased the concentrations of the TNFα, IL-6, IL-1β, and IL-10 proteins (*p* < 0.05) in the mice serum (Figure 9D–G). pBD114 also significantly increased (*p* < 0.05) the concentrations of the TNFα, IL-6, and IL-10 proteins in the mice serum (Figure 9D,E,G), and significantly decreased the LPS-induced concentrations of the IL-1β protein in the mice serum (Figure 9F) (*p* < 0.05).

**Figure 10:**
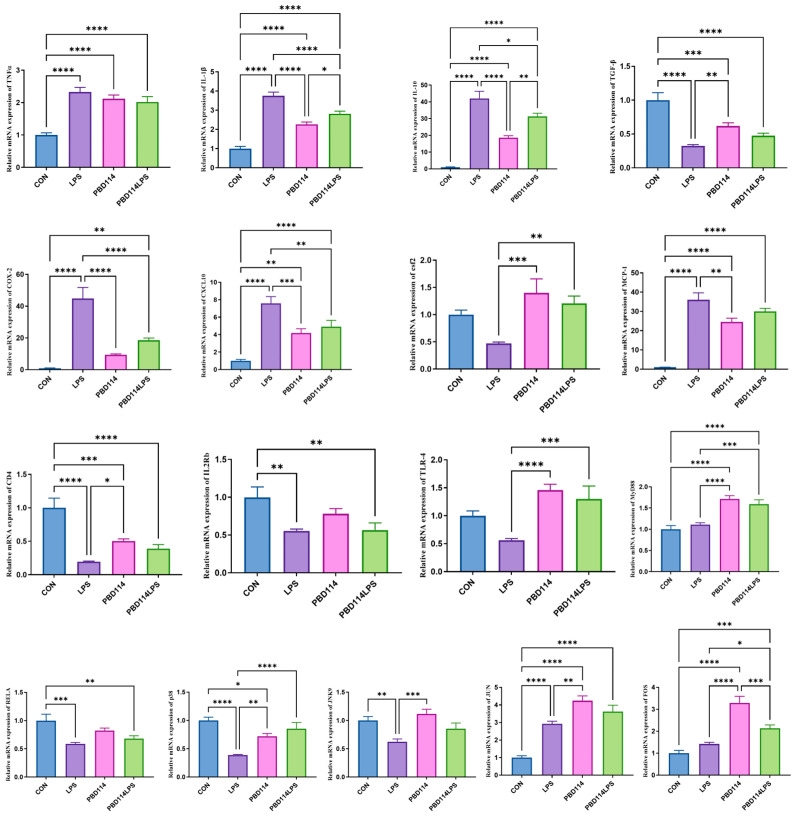
Effects of pBD114 on the gene expression level of the mouse spleens. The significance levels are * *p* < 0.05, ** *p* < 0.01, *** *p* < 0.001, and **** *p* < 0.0001.

The expression of inflammation-related genes in the mouse spleen was measured to investigate the effects of pBD114 on the inflammation response in the mice. As shown in Figure 10, by targeting the mouse spleen pro-inflammatory and anti-inflammatory factor-related genes, LPS significantly increased the mRNA levels of *TNFα*, *IL-1β*, and *IL-10* and significantly decreased the mRNA levels of *TGF-β* (*p* < 0.05), while pBD114 also considerably increased the mRNA levels of *TNFα*, *IL-1β*, *TGF-β*, and *IL-10* (*p* < 0.05); at the same time, pBD114 also significantly reduced the LPS-induced increase in the mRNA levels of *IL-1β* and *IL-10* (*p* < 0.05). For the mouse spleen chemokine-related genes, LPS significantly increased the mRNA levels of *COX-2*, *CXCL-10*, and *MCP-1* (*p* < 0.05), while pBD114 significantly increased the mRNA levels of *CXCL-10* and *MCP-1* (*p* < 0.05); meanwhile, pBD114 also significantly decreased LPS-induced increases in the *COX-2* and *CXCL-10* mRNA levels (*p* < 0.05) and significantly increased the LPS-induced decrease in the mRNA levels of *csf2* (*p* < 0.05). When targeting the inflammatory response-associated signaling pathway receptors, LPS significantly decreased the mRNA levels of *CD4* and *IL2Rb* (*p* < 0.05), and pBD114 significantly decreased the mRNA levels of *CD4* and significantly increased the mRNA levels of *TLR-4* (*p* < 0.05). When targeting inflammatory response-related transcription factor-related genes, LPS significantly decreased the mRNA levels of *RELA*, *p38*, and *JNK9* (*p* < 0.05) and significantly increased the mRNA level of *JUN*, and pBD114 significantly increased the mRNA levels of *p38*, *JUN*, and *FOS* (*p* < 0.05), and the mRNA level of LPS-induced *p38* (*p* < 0.05).

## 3. Discussion

The analysis of the porcine genome using bioinformatics methods in 2006 led to the initial discovery of pBD114, which is expressed in pig’s intestines, liver, spleen, lungs, and male reproductive tract [30]. The fact that pBD114 is expressed in several pig tissues is a harbinger of a diverse range of biological functions of pBD114. Our previous study utilized recombinant pBD114 obtained from the *E. coli* expression system to have weak activity against *E. coli* DH5α [31]. However, the recombinant pBD114 expressed in *E. coli* carries a tagged protein which may affect the spatial structure and activity of recombinant proteins. Therefore, in this study, pBD114 was prepared using chemical solid phase synthesis with >95% purity. Unfortunately, we also observed only weak antimicrobial activity by pBD114, with MIC values of 128 μg/mL against *Enterococcus faecalis* and *Salmonella typhimurium*, while all the other indicator bacteria had >256 μg/mL. The MIC values of pBD114 were consistent with the predicted results. Perhaps, pBD114 has no antibacterial activity, or maybe its antimicrobial activity can be further explored in future studies with natural pBD114. Nevertheless, an increasing amount of evidence indicates that the direct bactericidal activity of defensins in regulating the antibacterial immune response is not the only essential role of defensins in regulating host immune homeostasis [12]. The lack of anti-microbial activity of pBD114 may be compensated by immunomodulation, and some defensins have been shown to have such biological properties [32,33]. Similar to pBD2 [25] and pBD129 [20], pBD114 is not hemolytic or cytotoxic, which is also in agreement with the results of our previous study [31]. This may be the natural advantage of mammalian-sourced defensins.

The research on porcine defensins has shifted from anti-microbial activity to immunomodulation in recent years [13]. In particular, many studies have been conducted on porcine β-defensin 2 (pBD2), showing that pBD2 has antioxidant [25], intestinal barrier-enhancing [18], and anti-inflammatory activities [34]. Our previous study found that pBD114 can also inhibit the expression of inflammatory factors and alleviate the inflammatory injury of intestinal epithelial cells in mice caused by LPS [2]. However, can pBD114 regulate the inflammatory responses to macrophages? Macrophages play a critical role in initiating, maintaining, and resolving inflammation [35,36]. The well-studied murine macrophage cell line, RAW 264.7, is often used to initially screen natural products for bioactivity and predict their potential effect in vivo or on primary cells. The cell line response is used to evaluate the effective bioactivity of the product [37]. Therefore, RAW264.7 is used extensively to carry out in vitro screens for immunomodulators [38]. In contrast, the biological functions of defensins are related to the environment and cell type in which they are found [28]. Therefore, we used the RAW264.7 cells to investigate the effects of pBD114 on the inflammatory responses to RAW264.7 cells, which were treated with or without LPS. Our results showed that pBD114 significantly increased the levels of TNFα and IL-10 with a growing concentration of pBD114 and a prolonged treatment time, which were activated during the inflammatory response in RAW264.7 without LPS. However, when the inflammatory response of the RAW264.7 cells was activated by LPS, and then given the pBD114 treatment, pBD114 surprisingly again showed the inhibition of the inflammatory response, and the concentration of anti-inflammatory, active pBD114 was much lower than that of pro-inflammatory, active pBD114. The results were in agreement with that of a study on the pro- and anti-inflammatory effects of cathelicidin LL-37, which are concentration-dependent, i.e., the former are visible at >20 µg/mL, whereas the latter at 1–5 µg/mL [29]. The human cathelicidin LL 37 is transcribed from the CAMP gene in various cell types, including epithelial cells and many immune system cells. The effects of LL 37 are widespread, as this HDP is known to elicit a wide range of responses in a broad assortment of cell types. Similar to LL-37, pBD114 is also widely expressed in the intestines, liver, spleen, lungs, and male reproductive tract of pigs [30], indicating that the effects of pBD114 are also widespread.

Previous studies have shown that defensins exert pro-inflammatory and anti-inflammatory activities through signaling pathways such as NF-κB and MAPK [21,27,39]. However, these studies lacked the comprehensive and systematic investigation of the defensins’ pro-inflammatory and anti-inflammatory activities. RNA-seq is a technique used to study the overall transcription and transcriptional regulation laws of all the genes in a cell, which is also used to comprehensively and rapidly obtain the sequence information and expression information of almost all the transcripts of a specific cell or tissue in a certain state to accurately analyze the differences in gene expression, gene structure variability, the interaction networks between RNAs, and other important issues in life sciences [40]. Therefore, in this study, the effects of pBD114 on the inflammatory response of the RAW264.7 cells were analyzed using RNA-seq. The results showed that pBD114 significantly altered the expression of 1894 genes, of which 117 were up-regulated and 724 were down-regulated in the RAW264.7 cells in a normal physiological state. The differentially expressed genes were mainly enriched in the TNF, MAPK, and NF-kappa B signaling pathways which have been identified as pivotal regulators of the initiation and resolution of inflammation [41,42]. These results suggested that pBD114 activates the inflammatory response of RAW264.7 primarily through the TNF, MAPK, and NF-kappa B signaling pathways, which is consistent with the previous studies’ results [21,27,39]. PPI analysis showed that *Tnf*, *Kras*, *Jun*, *Src*, and *Mapk14* had the most interactions with the other proteins, and KDA analysis showed that *Bcl10*, *Bcl3*, *Cflar*, *Il1a*, *Dusp1*, *Traf1*, *Map2k7*, *Map3k8*, *Irf5* and *Ikbke* are the key driver genes in activating the inflammatory response of RAW264.7 by pBD114. *Bcl3* and *Traf1* are mainly involved in the TNF signaling pathway, which participates in negative intracellular signaling [43,44]. *Cflar* and *Bcl10* are primarily involved in the NF-kappa B signaling pathway, which participates in the B cell receptor signaling pathway and cell survival [45,46]. *Map2k7*, *Map3k8*, *Dusp1*, and *Il1a* are mainly involved in the MAPK signaling pathway, which activates ERK and JUK to promote proliferation, differentiation, and inflammation [47,48,49,50]. *Irf5* and *Ikbke* are mainly involved in the MyD88-independent pathway, which takes part in chemotactic and antiviral effects [51,52]. The above results indicate that pBD114 activates the inflammatory response through multiple signaling pathways and key genes such as TNF, MAPK, and NF-kappa B signaling pathways.

The effects of pBD114 on the inflammatory response of LPS-activated mouse monocyte macrophage RAW264.7 were analyzed using RNA-seq. However, pBD114 altered only 50 genes (up-regulated by 12 and down-regulated by 38) in the RAW264.7 cells with the inflammatory response activated by LPS. KEGG enrichment analysis showed that these genes were mainly enriched in the cytokine–cytokine receptor interaction, Viral protein interaction with cytokine and cytokine receptor, Hematopoietic cell lineage, and Th17 cell differentiation. PPI analysis showed that *Stat1* and *Cxcl10* had the most interactions with the other proteins. Further KDA analyses showed that *Stat1*, *Csf2,* and *Il1b* are the key driver genes. These results suggest that the inhibition of RAW264.7-activated inflammatory responses by pBD114 is not strong, unlike HBD3 and pBD2. There was a substantial effect of HBD3 on TLR4 activation; a total of 5494 genes compared with 1779 genes were differentially expressed between the macrophages stimulated with KLA or KLA and hBD3, respectively [53]. Similarly, there were 812 DEGs between the *E. coli* and *E. coli* + pBD2 groups, including 431 significantly up-regulated genes and 381 significantly down-regulated genes [54].

To further verify the effects of pBD114 on inflammatory response, we carried out an in vivo assay of the effects pBD114 on the inflammatory response in mice. The results showed that pBD114 could activate the inflammatory response in mice and inhibit the inflammatory response to some extent after LPS induced it, which is consistent with the results of studies on human and porcine defensins [20,27,55]. As shown by the previous findings and our own, the effect of defensins on the inflammatory response goes far beyond simply acting as an inflammatory response modulator via a singular receptor or linear signaling. They have a multifaceted effect on multiple pathways in a cell and organism, and the effect varies in different environments. Therefore, determining the mechanisms of the defensins’ action on cells or organisms in characteristic environments is essential for revealing the defensins’ functions.

## 4. Materials and Methods

### 4.1. Chemical Solid-Phase Synthesis of pBD114

The pBD114 mature peptide amino acid sequence was obtained from NCBI entry number NP_001123445.1 and was synthesized using the solid-phase method with minor modifications [56]. The reaction conditions for pBD114 extension were as follows: 3-fold excess of Fmoc protecting group amino acids; 3-fold excess of HBTU/HOBT as coupling reagents; 6-fold excess of N, N-Diisopropylethylamine, and DMF as a solvent. Deprotections were performed using a solution of 20% (*v*/*v*) piperidine in DMF. Finally, the pBD114 was cleaved with TFA:H_2_O:TIS = 95:2.5:2.5 (*v*/*v*), and purified using RP-HPLC (Agilent Santa Clara, CA, USA) with a C18 column (21.2 × 250 mm). Purity was analyzed using RP-HPLC (Agilent Santa Clara, CA, USA) with a C18 column (4.6 × 250 mm). The molecular mass was confirmed using electrospray ionization-mass spectrometry (EI-MS, LCMS-2020, Shimadzu, Kyoto, Japan).

### 4.2. Minimal Inhibitory Concentration

The minimal inhibitory concentration (MIC) of pBD114 was determined using the microtiter broth dilution method [57]. *Staphylococcus aureus* ATCC 29213, *Staphylococcus aureus* ATCC 43300, *Enterococcus faecalis* ATCC 29212, *Escherichia coli* ATCC 25922, *Pseudomonas aeruginosa* ATCC 27853, *Salmonella typhimurium* ATCC 13311, and *Klebsiella pneumoniae* ATCC 13883 were grown to 0.4 OD_600nm_ at 37 °C in MH. The target cell culture was diluted to 1 × 10^5^ CFUs/mL with MH, respectively. Totals of 100 μL of pBD114 and 100 μL of cell suspension were added into each well. The activity of pBD114 was tested at concentrations of 0.25, 1, 2, 4, 16, 32, 64, 128, and 256 μg/mL, and all the assays were tested in triplicated. Bacterial plates were incubated at 37 °C for 16 h, and the absorption of cell culture was recorded at 600 nm. MIC was defined as the lowest concentration of peptide at which there was no change in the optical density.

### 4.3. Hemolytic Activity Assay

The hemolytic activity of pBD114 was measured spectrophotometrically using a hemoglobin release assay [18]. Fresh pig blood was collected to prepare erythrocytes by centrifugation at 1500 rpm for 10 min at room temperature. The erythrocytes were washed three times warmly with PBS (pH 7.2) and resuspended at 4% (*v*/*v*) in PBS (pH 7.2). A total of 4% erythrocytes was incubated with 1, 4, 16, 64, and 256 μg/mL pBD114 and melittin for 1 h at 37 °C, and then centrifuged at 1000 rpm for 5 min. A total of 3 mL supernatant was added to a 2 cm quartz cuvette, and the absorbance was measured at 414 nm with a UV-1100 spectrophotometer (Mapada, Shanghai, China). No hemolysis and 100% hemolysis were determined in PBS and Triton X-100, respectively.

### 4.4. Cell Culture and Treatment

The mouse mononuclear macrophage RAW264.7 cells were cultured in a 75 cm^2^ cell culture flask in DMEM-F12 with 10% FBS, 100 U/mL penicillin, and 100 μg/mL streptomycin. The cells were cultured in an incubator with sufficient humidity at 37 °C and 5% CO_2_. The effects of pBD114 on the inflammatory response of the RAW264.7 cells were tested as follows:

(1) Cytotoxicity of pBD114: The cells were inoculated into 96-well culture plates and cultured until the fusion reached 50%. The cells were treated with pBD114 at final concentrations of 0, 1, 4, 16, 64, and 256 μg/mL for 24 h. CCK-8 detected the cell activity of pBD114.
(2) Effects of pBD114 on inflammatory response in macrophages under normal physiological conditions: The cells were inoculated into 24-well culture plates and cultured until the fusion reached 80%, and then treated with 10 and 100 μg/mL pBD114 for 1, 3, 6, and 12 h. Cell culture medium was collected to detect the TNF-α (R&D Systems, DY410, MN, USA) and IL-10 (R&D Systems, M1000B, MN, USA) protein concentrations using ELISA.
(3) Optimization of LPS treatment time and concentration: The cells were inoculated into a 24-well culture plate and cultured until the fusion reached 80%, and then treated with 1, 5, 10, and 20 μg/mL LPS and 50 μg/mL pBD114f for 0.5, 1, 2, and 4 h; the cell culture medium was collected, and the TNF-α protein concentration was detected using ELISA.
(4) Effects of pBD114 on the inflammatory response to macrophages under inflammatory activation: The cells were inoculated into a 24-well culture plate and cultured until the fusion reached 80%, and then treated with 1, 5, 10, 20, and 50 μg/mL pBD114 and 10 ng/mL LPS for 2 h; the cell culture medium was collected, and the TNF-α protein concentration was detected using ELISA.
(5) Samples of RNA-seq: The RAW264.7 cells were inoculated into a 6-well plate and cultured until the fusion reached 80%. The cells were treated with or without pBD114 for 12 h, and 10 ng/mL LPS-induced cells were simultaneously treated with or without 10 μg/mL pBD114 for 2 h. The concentration of TNF-α and IL-10 protein in the culture solution was detected using ELISA. The cells were collected for RNA-seq.

### 4.5. RNA-Seq and Data Analysis 

Four biological replicates of each sample were submitted to BGI Genomics (The Beijing Genomics Institute, Beijing, China) for RNA extraction, library construction, and RNA sequencing. High-throughput sequencing was performed using the DNBSEQ platform (BGI, Shenzhen, China) with 150 bp paired-end reads [58]. Data analysis was conducted using the Dr. Tom system from BGI http://report.bgi.com (accessed on 23 November 2022). Differentially expressed genes were identified based on an adjusted *p*-value < 0.05 and fold-change > 2. RT-qPCR further validated the results.

### 4.6. Animal Trials

A mouse assay was performed to investigate the effects of pBD114 on the inflammatory response in the animals; a total of 40 C57BL/6 mice (7 weeks) were randomly allocated to four groups: CON, treated without pBD114 and LPS; PBD114, treated with 10 mg/kg pBD114 and without LPS (Sigma-Aldrich, St Louis, MO, USA); LPS, treated without pBD114 and with 1 mg/kg LPS; and PBD114LPS, treated with 1 mg/kg pBD114 and with 1 mg/kg LPS. pBD114 and LPS were intraperitoneally injected using a 1 mL insulin syringe (Braun, Melsungen, Germany) within 200 μL PBS. After 12 h, all mice were sacrificed using carbon dioxide anesthesia, their livers and serum were collected for analysis, and their weight was measured.

### 4.7. Quantitative Real-Time PCR Analysis

Total RNA was extracted from the tissues of the spleen using the Trizol reagent (Takara, Beijing, China), followed by reverse transcription using the High Capacity cDNA Archive kit (Takara, Beijing, China), according to the manufacturer’s protocols. Real-time PCR was carried out using the ABI 7500 real-time PCR system (Applied Biosystems, Waltham, MA, USA) involving SYBR Green. The primer sequences used for the mRNA determined are listed in Table S7. The relative abundance of a target gene was calculated using the 2^−∆∆Ct^ method [59].

### 4.8. Statistical Analysis

Statistical tests were performed using GraphPad Prism 9 software (GraphPad, La Jolla, CA). The results are shown as the mean ± SE. The statistical significance of the data was determined using the T-test (unpaired) or one-way ANOVA and Dunnett’s multiple range test (*p* < 0.05). In all cases, a *p*-value < 0.05 was considered to be statistically significant (* *p* < 0.05, ** *p* < 0.01, and *** *p* < 0.001).

## 5. Conclusions

In vitro and in vivo tests have shown that high concentrations of pBD114 activate the inflammatory response, and low concentrations inhibit the activated inflammatory response. RNA-seq analyses indicated that pBD114 activates the inflammatory response through the *Bcl10*, *Bcl3,* and immune- and signal- transduction-related signaling pathways. pBD114 inhibits the activated inflammatory response through the *Stat1*, *Csf2*, and “Cytokine–cytokine receptor interaction” signaling pathway. Although the inflammatory response is a rapid and complex physiological reaction to noxious stimuli, this study found that pBD114 plays an important role in the inflammatory response mainly by acting on the genes related to immunity, signal transduction, signaling molecules, and interactions. However, there are still some limitations to this study, such as not validating the role of pBD114 in modulating the inflammatory response using more cell lines. In addition, it would be more meaningful to investigate the biological functions of pBD114 with primary cells or cell lines from pigs. In conclusion, this study provides a certain theoretical basis for research on and the application of defensin.

## Data Availability

Data is contained within the article and Supplementary Material.

## Supplementary Materials

The supporting information can be downloaded at: https://www.mdpi.com/article/10.3390/ijms25021016/s1.
