# Supplementary material for: Porcine β-Defensin 114: Creating a Dichotomous Response to Inflammation
Source: Int J Mol Sci. 2024 Jan 13;25(2):1016. doi: 10.3390/ijms25021016 (PMC10816359; doi:10.3390/ijms25021016)
Supplement: Supplementary file 1 [file ijms-25-01016-s001.zip › ijms-2787559-supplementary.pdf]

International Journal of *Molecular Sciences*

MDPI

A

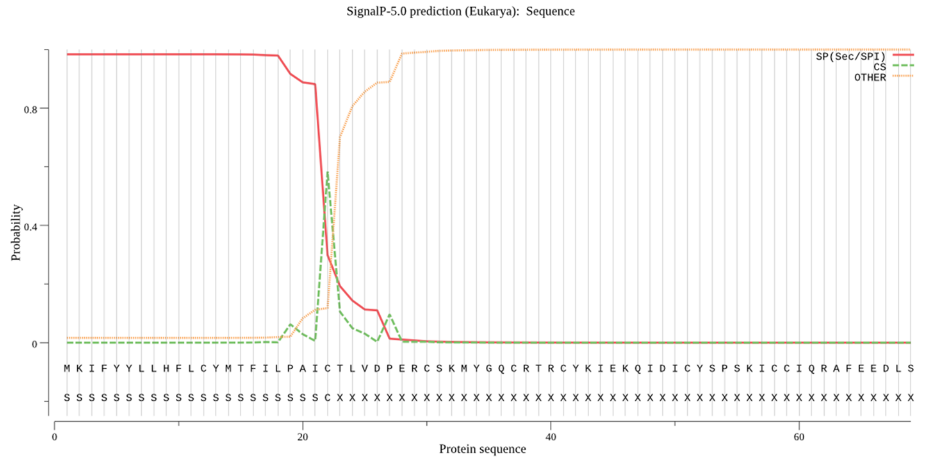

Prediction: Signal peptide (Sec/SPI)

Cleavage site between pos. 22 and 23: AIC-TL. Probability: 0.5835

Protein type	Signal Peptide (Sec/SPI)	Other
Likelihood	0.9836	0.0164

MKIFYYLLHFLCYMTFILPAICTLVDPERCSKMYGQCRTRCYKIEKQIDICYSPSKICCIQRAFEEDLS

B

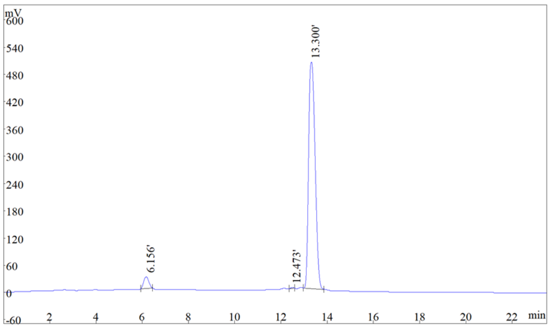

C

Rank	Time	Conc.	Area	Height
1	6.16	3.96	370473	25345
2	12.47	0.25	23643	2692
3	13.30	95.79	8957889	498322
Total		100	9352005	526359

D

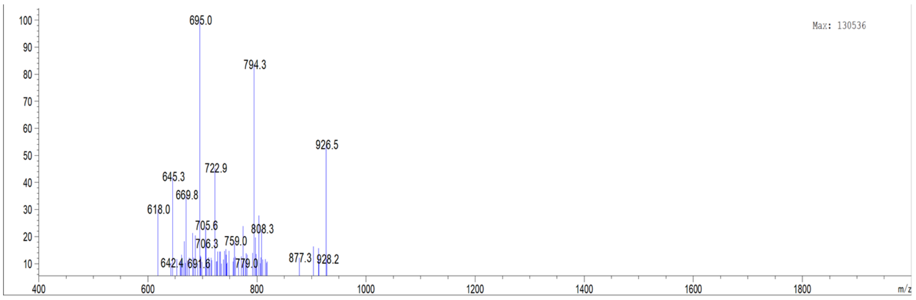

Supplementary Figure S1. Chemical solid phase synthesis and quality inspection of pBD114. (A) The signal cleavage site of pBD114's precursor protein was determined by SignalP-5.0 to be between the 22nd and 23rd amino acids. The purity of pBD114 synthesized by chemical solid phase was higher than 95% (C) by HPLC (B). The molecular mass was confirmed by electrospray ionization-mass spectrometry (D).

ID	sequence	AMP	Anti-parasitic	Anti-viral	Anti-cancer	Targeting mammals	Anti-fungal	Targeting Gram positive bacteria	Targeting Gram negative bacteria
pBD114	TLVDPERCSKMYGQCRTRCYKIEKQIDICYSPSKICCIQRAFEEDLS	0.669	0.0	0.1417	0.0104	0.0333	0.7084	0.2833	0.3115

Supplementary Figure S2. The bioactivity of pBD114 predicted online with AMPfun. Green means positive prediction, pink means negative prediction.

Table S1. Minimum inhibitory concentration value of pBD114 against various indicator bacteria.

Item	PBD114, μg/mL	Polymyxin, μg/mL	Vancomycin, μg/mL
Staphylococcus aureus ATCC 29213	> 256	256	< 0.25
Staphylococcus aureus ATCC 43300	> 256	128	< 0.25
Enterococcus faecalis ATCC 29212	128	> 256	1
Escherichia coli ATCC 25922	> 256	< 0.25	32
Pseudomonas aeruginosa ATCC 27853	> 256	< 0.25	> 256
Salmonella typhimurium ATCC 13311	128	0.5	64
Klebsiella pneumoniae ATCC 13883	> 256	< 0.25	128

Peptide Sequence	Mutation Position	PROB score	Hydrophobicity	Hydropathicity	Hydrophilicity	Mol wt
TLVDPERCSKMYGQCRTRCYKIEKQIDICYSPSKICCIQRAFEEDLS	No Mutation	0.43	-0.27	-0.53	0.31	5553.06

Supplementary Figure S3. Hemolysis of pBD114 predicted online with HemoPI. PROB score is the normalized SVM score and ranges between 0 and 1, i.e. 1 very likely to be hemolytic, 0 very unlikely to be hemolytic.

Table S2. The quality control information for transcriptome samples

Sample	Total Raw Reads, M	Total Clean Reads, M	Total Clean Bases, Gb	Clean Reads Q20, %	Clean Reads Q30, %
LPS-1	23.92	23.83	1.19	97.82	93.66
LPS-2	23.92	23.83	1.19	97.86	93.73
LPS-3	23.92	23.82	1.19	97.87	93.78
LPS-4	22.24	22.15	1.11	97.76	93.47
MOCK-1	23.92	23.83	1.19	97.77	93.53
MOCK-2	23.92	23.83	1.19	97.83	93.72
MOCK-3	23.92	23.81	1.19	97.81	93.60
MOCK-4	23.92	23.83	1.19	97.84	93.75
PBD114-1	23.92	23.70	1.18	97.70	93.28
PBD114-2	23.92	23.67	1.18	97.47	92.69
PBD114-3	23.92	23.66	1.18	97.77	93.52
PBD114-4	23.92	23.69	1.18	97.47	92.73
PBD114LPS-1	23.92	23.86	1.19	97.40	92.22
PBD114LPS-2	23.92	23.85	1.19	97.46	92.40
PBD114LPS-3	22.91	22.84	1.14	97.59	92.69
PBD114LPS-4	21.26	21.20	1.06	98.08	93.98

Table S3. The results about the comparison of clean reads with reference genome.

Sample	Total Clean Reads (M)	Total Mapping (%)	Uniquely Mapping (%)
MOCK-1	23.83	90.18	68.90
MOCK-2	23.83	90.01	69.11
MOCK-3	23.82	89.59	69.41
MOCK-4	22.15	89.44	69.17
LPS-1	23.83	90.19	69.10
LPS-2	23.83	90.42	68.89
LPS-3	23.81	90.69	69.02
LPS-4	23.83	90.51	68.85
PBD114-1	23.70	89.81	69.28
PBD114-2	23.67	88.51	69.34
PBD114-3	23.66	89.53	69.10
PBD114-4	23.69	88.57	69.70
PBD114LPS-1	23.86	89.70	69.20
PBD114LPS-2	23.85	90.30	69.09
PBD114LPS-3	22.84	89.93	69.60
PBD114LPS-4	21.20	91.61	69.16

Table S7. The primer sequences

Genes	ACCESSION	Primer sequence	Product size	Tm/°C
β-actin	NM_001272041.1	AAGCGAGGTATCCTGACCCT<br>GATGTCGCGCACAATCTCAC	456	60
IL-10	NM_010548.2	GCAGAGAAGCATGGCCCAGAA<br>TGGCCTTGTAGACACCTTGGTCTT	181	60
TGF-β	NM_011577.2	CGAACCCCCATTGCTGTCCC<br>CGTTTGGGGCTGATCCCGT	294	60
CD4	NM_013488.3	CCAACAGCGCCAGGCA<br>AGTGTCTGAAACGCAGAGGG	295	60
IL2Rb	NM_008368.4	GCAGGGAACATCTCGACACAA<br>CAGTGTCGCAGGTTCGACTT	277	60
TLR-4	NM_021297.3	CGCTGCCACCAGTTACAGAT<br>CTTCAAGGGGTTGAAGCTCAG	263	60
MyD88	NM_010851	AGCAGAACCAGGAGTCCGAGAAG<br>GGGCAGTAGCAGATAAAGGCATCG	148	60
RELA	NM_009045	AGACCCAGGAGTGTTCACAGACC<br>GTCACCAGGCGAGTTATAGCTTCAG	141	60
P38	NM_011951	CTGGCTCGGCACACTGATGATG<br>GCCCACGGACCAAATATCCACTG	120	60
JNK9	NM_207692	ACTATCGGGCTCCAGAAGTCATCC<br>ATCACACAACCTTTCACCAGCTCTC	103	60
JUN	*NM_010591*	CTTCTACGACGATGCCCTCAACG	105	60

		GCCAGGTTCAAGGTCATGCTCTG		
FOS	NM_010234	AAGACCGTGTCAGGAGGCAGAG<br>CAGCCATCTTATTCCGTTCCCTTCG	115	60
TNFα	NM_013693.3	TGTAGCCCACGTCGTAGCAA<br>TGTAGCCCACGTCGTAGCAA	216	60
IL-1β	NM_008361.4	TGCCACCTTTTGACAGTGATG<br>AAGGTCCACGGGAAAGACAC	220	60
Icam1	NM_010493.3	CTGGGCTTGGAGACTCAGTG<br>CCACACTCTCCGGAAACGAA	175	60
Ptgs2	NM_011198.5	CATCCCCTTCCTGCGAAGTT<br>CATGGGAGTTGGGCAGTCAT	178	60
Csf2	NM_009969.4	CTGGCCCCATGTATAGCTGA<br>CTGGCCCCATGTATAGCTGA	170	60
Cxcl2	NM_009140.2	CTGGCCCCATGTATAGCTGA<br>CTGGCCCCATGTATAGCTGA	184	60
Cxcl10	NM_021274.2	GAGAGACATCCCGAGCCAAC<br>TCAACACGTGGGCAGGATAG	228	60
Ccl2	NM_011333.3	AGGTGTCCCAAAGAAGCTGT<br>AGGTGTCCCAAAGAAGCTGT	163	60
